# The Role of *Grifola frondosa* Polysaccharide in Preventing Skeletal Muscle Atrophy in Type 2 Diabetes Mellitus

**DOI:** 10.3390/life14070784

**Published:** 2024-06-21

**Authors:** Ying She, Yun Ma, Pei Zou, Yang Peng, Yong An, Hang Chen, Peng Luo, Shaofeng Wei

**Affiliations:** 1 The Key Laboratory of Environmental Pollution Monitoring and Disease Control, Ministry of Education, Guizhou Provincial Engineering Research Center of Ecological Food Innovation, Collaborative Innovation Center for Prevention and Control of Endemic and Ethnic Regional Diseases Co-Constructed by the Province and Ministry, School of Public Health, Guizhou Medical University, Guiyang 561113, China; 2 State Key Laboratory of Functions and Applications of Medicinal Plants, Guizhou Medical University, Guiyang 550025, China

**Keywords:** type 2 diabetes mellitus, skeletal muscle atrophy, *Grifola frondosa* polysaccharide, gut–muscle axis, inflammation, molecular docking

## Abstract

Type 2 diabetes mellitus (T2DM) is a burgeoning public health challenge worldwide. Individuals with T2DM are at increased risk for skeletal muscle atrophy, a serious complication that significantly compromises quality of life and for which effective prevention measures are currently inadequate. Emerging evidence indicates that systemic and local inflammation stemming from the compromised intestinal barrier is one of the crucial mechanisms contributing to skeletal muscle atrophy in T2DM patients. Notably, natural plant polysaccharides were found to be capable of enhancing intestinal barrier function and mitigating secondary inflammation in some diseases. Herein, we hypothesized that *Grifola frondosa* polysaccharide (GFP), one of the major plant polysaccharides, could prevent skeletal muscle atrophy in T2DM via regulating intestinal barrier function and inhibiting systemic and local inflammation. Using a well-established T2DM rat model, we demonstrated that GFP was able to not only prevent hyperglycemia and insulin resistance but also repair intestinal mucosal barrier damage and subsequent inflammation, thereby alleviating the skeletal muscle atrophy in the T2DM rat model. Additionally, the binding free energy analysis and molecular docking of monosaccharides constituting GFP were further expanded for related targets to uncover more potential mechanisms. These results provide a novel preventative and therapeutic strategy for T2DM patients.

## 1. Introduction

Type 2 diabetes mellitus (T2DM) is characterized by metabolic disorders such as hyperglycemia and insulin resistance (IR) [1,2], which has become one of the fastest-growing metabolic diseases in the world [3]. It is estimated that the number of patients with impaired glucose tolerance worldwide will be 454 million in 2030 and 548 million in 2045 [4], which presents substantial challenges to the quality of life of individuals and socio-economic burden.

Skeletal muscle atrophy, a common complication in patients with long-standing and poorly controlled T2DM [5], contributes to the decline of their quality of life, increased fracture risk, and even mortality [6]. Preventing skeletal muscle atrophy is crucial for enhancing the quality of life and mitigating complications in individuals with T2DM. But to date, no effective treatment has been developed for skeletal muscle atrophy. Exercise is widely considered to be the most effective treatment for skeletal muscle atrophy, which is unfortunately not suitable for all patients [7].

Chronic inflammation is a prominent characteristic of T2DM [8]. Long-term systemic inflammation not only aggravates the typical insulin resistance of T2DM, but also leads to skeletal muscle atrophy by reducing muscle protein synthesis and increasing protein degradation, which also represents a primary mechanism underlying the skeletal muscle atrophy in this condition [9,10]. Emerging evidence indicates that the systemic chronic inflammation observed in patients with T2DM mainly results from intestinal barrier dysfunction during metabolic dysregulation [7]. The impaired gut mucosal barrier facilitates the entry of luminal bacteria products and metabolites, especially lipopolysaccharides (LPS), into the circulation, triggering a state of inflammation in various organs, as well as in the skeletal muscles [11,12], where the inflammation-sensitive nuclear factor κB (NF-κB) signal transducer may contribute to muscle atrophy in T2DM [10]. Compared to pathogen-free mouse skeletal muscle, germ-free mouse skeletal muscle showed atrophy [13]. The abnormality of intestinal flora in patients with sarcopenia is related to inflammatory response, and the composition of intestinal flora is significantly different from that of normal people [14]. More importantly, animal studies have found that transplantation of gut microbiota from pathogen-free mice to germ-free mice can increase skeletal muscle mass and reduce markers of muscle atrophy [13], and probiotic supplementation inhibited inflammatory signaling pathways such as NF-κB and improved muscle atrophy [15,16]. In population trials, the probiotic *Lactobacillus plantarum* supplementation in the elderly was beneficial to muscle quality [17], which may be due to reducing muscle atrophy by reducing intestinal permeability [18]. These studies highlight the gut–muscle axis as a potential therapy target for combating muscle dysfunction in T2DM. 

Natural plant polysaccharides, as a significant category of prebiotics, have exhibited therapeutic potential in managing diabetes and inflammatory bowel disease [19,20]. Dendrobium polysaccharide was found to be capable of repairing the intestinal barrier and reducing inflammation through the LPS/TLR4/TRIF/NF-κB axis [21]. Fructus mori polysaccharide was demonstrated to relieve hyperglycemia, insulin resistance, endotoxemia, and intestinal inflammation by inhibiting the activation of the TLR4/MyD88/NF-κB pathway [22]. As an important natural plant polysaccharide, *Grifola frondosa* polysaccharide (GFP) was previously reported to acquire the ability to prevent hyperlipidemia and improve type 2 diabetes mellitus by regulating specific intestinal microflora [23,24]. Moreover, GFP could alleviate diabetic nephropathy by blocking the TLR4/NF-κB pathway [25]. Moreover, according to previous research findings of our research group, GFP also decreased the expression of pro-inflammatory cytokines tumor necrosis factor (TNF)-α reduced colon injury in ulcerative colitis mice [26]. However, whether GFP has the capability of preventing skeletal muscle atrophy in T2DM remains unknown.

Collectively, given the increasing evidence indicating the potent effects of GFP on T2DM, we proposed that GFP can not only regulate the symptoms related to hyperglycemia but enhance intestinal barrier function, inhibit systemic and local inflammation, and eventually prevent skeletal muscle atrophy in T2DM. In this study, we investigated the aforementioned hypothesis utilizing a T2DM rat model.

## 2. Materials and Methods

### 2.1. Preparation of GFP

The extraction and purification of GFP were carried out according to the conditions of the previous experiments of our group [26]. In brief, the crude GFP was obtained from *Grifola frondosa* powder through ultrasound-assisted extraction, which was then purified with polyamide, DEAE-52 cellulose ion exchange column, and Sephadex G-100 separately. Then, purified GFP was prepared for the following experiments.

### 2.2. Animals

Fifteen adult male SD rats, aged 7 weeks and weighing from 260 g to 280 g, were purchased from the Animal Research Center of Guizhou Medical University. The animals were kept under conventional light conditions (12 h:12 h dark/light cycle) and constant temperature (22 ± 1 °C) with free drinking water and feed. All procedures and animal experiments in this study were approved by the Experimental Animal Ethics Committee of the Guizhou Medical University, China (No. 202303090).

**Figure 1:**
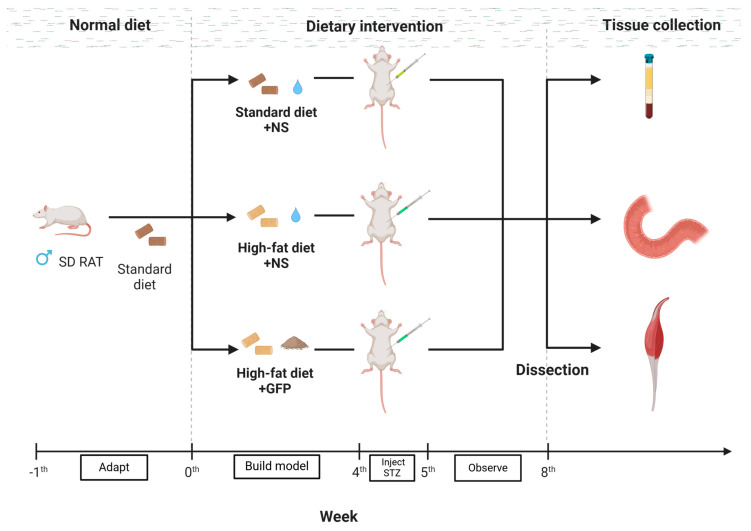
The T2DM rat model was established, and the tissues from each group were collected (the schematic drawing was created with BioRender.com (accessed on 24 April 2024)).

After 1 week of acclimation, the rats were randomly divided into three groups (*n* = 5), namely the control group (NC, *n* = 5), the type 2 diabetes group (T2DM, *n* = 5), and the *Grifola frondosa* polysaccharide prevention group (GFP-P, *n* = 5). Rats in the NC group were fed a standard diet (HUANYU BIO, Anyang, China), while the T2DM group and GFP-P rats were fed with high-fat diet (HD001, Beijing Botai Hongda Biotechnology, Beijing, China) consisting of 45% fat, 35% carbohydrates, and 20% protein. Rats in the GFP-P group were gavaged with normal saline proportioned GFP (1.5 g/kg), while the ones in the NC group and T2DM group were gavaged with corresponding volumes of normal saline. The administration of gavage in the three groups was carried out at specific time points every day. After a 4-week high-fat diet with or without GFP gavage, streptozotocin (STZ) solution was prepared with sodium citrate buffer and was intraperitoneally injected (35 mg/kg) in the GFP-P group and T2DM group. A 4-week standard diet and 0.9% equal dose sodium chloride solution was used in the NC group. The fasting blood glucose (FBG) of rats in the T2DM group was >11.1 mmol/L and remained stable within 7 days, and the “three more and one less” characteristics, namely polyuria, polydipsia, hyperphagia, and weight loss, were observed, which were regarded as a successful T2DM model. In the 8th week, after oral administration, all rats fasted overnight and were anesthetized by a sodium pentobarbital solution. Blood was collected from the femoral artery and stored in a −20 °C refrigerator after centrifugation. Proximal colon and gastrocnemius muscles were taken. Part of the colon and gastrocnemius muscle were fixed in paraformaldehyde and skeletal muscle fixative solution, respectively. The remaining samples were quickly passed through liquid nitrogen and stored in a −80 °C refrigerator. The schematic drawing depicts the timeline of the T2DM rat model establishment (Figure 1).

### 2.3. Body Weight, Blood Glucose Level, and Glucose Tolerance Test

The body weight of rats was measured at 9:00 a.m. every Monday, and the FBG level was monitored by tail vein blood sampling to evaluate the change of blood glucose level. In the 8th week, the rats fasted for 12 h, and all rats were injected with glucose solution at a dose of 2 g/kg for the intraperitoneal glucose tolerance test (GTT). Tail vein blood samples were collected at 0, 30, 60, 90, and 120 min, and the blood glucose level was detected with a portable blood glucose monitor (NoCoding1, i-SENs, Seoul, South Korea). The curve of blood glucose concentration with time was drawn, and then the area under the curve (AUC) was calculated.

### 2.4. Enzyme-Linked Immunosorbent Assay (ELISA)

ELISA Kits were used to determine the levels of endotoxin LPS (Westang, Shanghai, China), insulin INS (JYM0620Ra, JI YIN MEI, Wuhan, China), diamine oxidase DAO (jym0013ra, JI YIN MEI, Wuhan, China) and D-lactate D-LA (JYM0693Ra, JI YIN MEI, Wuhan, China) in rat serum, following each corresponding protocol from the manufacturer. The insulin resistance index (HOMA-IR) was calculated as (FBG level × INS level)/22.5.

### 2.5. Real-Time Quantitative PCR (RT-qPCR)

Total RNA was extracted from the rat colon and gastrocnemius muscle using Trizol reagent (Thermo Fisher, Waltham, MA, USA). The cDNA was reverse transcribed using gDNA digester plus (Yeasen, Shanghai, China). Then Universal blue qPCR SYBR Green master (Yeasen, Shanghai, China) was used for RT qPCR detection to detect the mRNA expression level. The relative expression levels between samples were evaluated using the ΔΔCt method. Primer information is shown in Table 1.

### 2.6. Histopathological Analysis (HE)

The fixed rat colon and gastrocnemius muscles were dehydrated and embedded in paraffin. Paraffin-embedded tissues were then cut into 4 μM- and 2 μM-thick sections, which were pathologically stained with hematoxylin eosin (Servicebio, Wuhan, China). Images were obtained using a microscope (Nikon, Tokyo, Japan), and the cross-sectional area (CSA) of gastrocnemius muscle fiber cells was quantitatively determined using ImageJ Fiji software.

### 2.7. Immunohistochemistry (IHC)

The colon and muscle tissue sections were dewaxed and repaired by heat, then inactivated with endogenous peroxidase and sealed with a blocking buffer at room temperature. The corresponding first antibodies were then incubated, including ZO-1 (AF5145, Affinity, Liyang, China), Occludin (DF7504, Affinity, Liyang, China), TNF-α (BA0131, Boster, Guangzhou, China), NF-κB p65 (BS-0465R, Boster, Guangzhou, China), FBXO32 (67172-1-1g, Proteintech, Wuhan, Hubei, China), and TRIM63 (55456-1-AP, Proteintech, Wuhan, China) overnight at 4 °C. On the second day, tissue sections were incubated with anti-mouse/rabbit secondary antibodies (PK10006, Proteintech, Wuhan, China) at room temperature, then stained with diaminobenzidine; then, sections were re-stained with hematoxylin, differentiated in differentiation solution, dehydrated, and sealed. We used a microscope (Nikon, Tokyo, Japan) to obtain images, and each sample took 3 images. The protein expression level in IHC was quantitatively analyzed by ImageJ Fiji software.

### 2.8. Western Blotting (WB)

The total protein of gastrocnemius muscle tissues was extracted by RIPA lysis method (BL509A, Biosharp, Chongqing, China). The total protein was quantified by the BCA protein detection kit (P0009, Beyotime, Shanghai, China), and denatured by adding 5× loading buffer. The protein was separated by polyacrylamide gel electrophoresis (SDS-PAGE) and transferred to the polyvinylidene fluoride (PVDF, ISEQ00010, Sigma-Aldrich, St. Louis, MO, USA) membrane. After being washed with TBST (TBS + Tween), the membrane was sealed in skimmed milk and incubated overnight with a suitable primary antibody at 4 °C. The primary antibody included TNF-α (Proteintech, Wuhan, China), NF-κB p65 (Affinity, Liyang, China), FBXO32 (67172-1-lg, Proteintech, Wuhan, China), TRIM63 (55456-1AP, Proteintech, Wuhan, Hubei, China), and β- Tubulin (T0023, affbiotech, Shanghai, China). Then, after washing with TBST, the PVDF membrane was incubated with the secondary antibody (Proteintech, Wuhan, China) at room temperature. The band was revealed by a chemiluminescence detection system (Beyotime, Shanghai, China) and exposed in the fluorescence image analysis system (5200multi, Tanon, Shanghai, China). The results were scanned by Gel-Pro analyzer 4 software, and the integrated optical density (IOD) of the target protein was analyzed.

### 2.9. Molecular Docking (MD)

The 2D structure of monosaccharide was obtained by PubChem database (http://pubchem.ncbi.nlm.nih.gov/ (accessed on 28 April 2024)). The 2D structure was inputted into Chem Office 20.0 software to make its 3D structure and saved as mol2 file. Then, the crystal structure with high resolution and screened by RCSB Protein Data Bank database (http://www.rcsb.org/ (accessed on 28 April 2024)) was used as the molecular docking receptor. The protein was dehydrated and dephosphorized by PyMOL 2.6.0 software and saved as a PDB file. The energy of the compound was minimized by Molecular Operating Environment 2019 software, the target protein was pretreated, and the active pocket was searched. Finally, we ran MOE 2019 for molecular docking, and the number of operations was 50 times. The binding activity of the two was evaluated according to the binding energy, and the results were visualized by PyMOL 2.6.0 and Discovery studio 2019 software [27].

### 2.10. Statistical Analysis Method

All data were analyzed by SPSS 26.0 statistical software and mapped by GraphPad Prism 9 software. The one-way ANOVA test was used for the comparison between the mean values of the diversity, the LSD test was used for the homogeneous variance, and Tamhane’s T2 test was used for the inhomogeneous variance. Pearson or Spearman was used to perform the correlation analysis according to the results of normal distribution examination. When *p* < 0.05, it was considered that the difference between groups was statistically significant. The data are reported as mean ± SD. 

## 3. Results

### 3.1. GFP Prevented Hyperglycemia, Insulin Resistance, and Weight Loss in T2DM Rats

**Figure 2:**
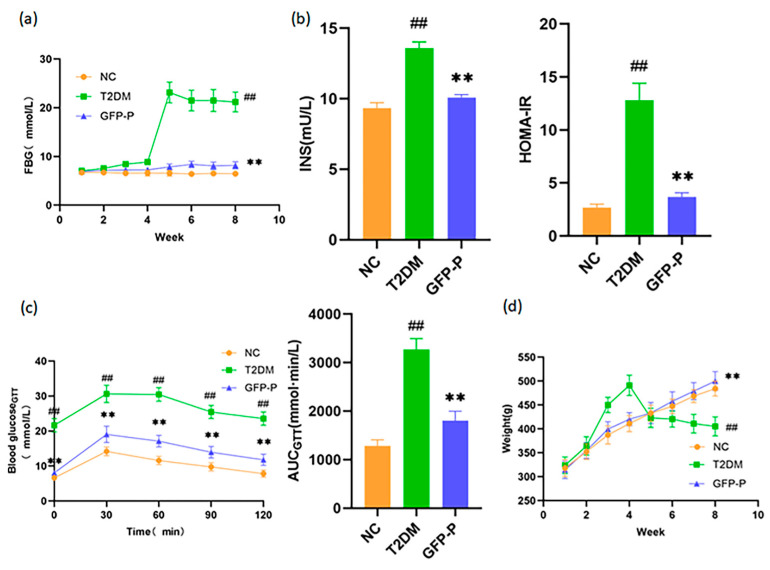
GFP prevented hyperglycemia, insulin resistance, and weight loss in T2DM rats. (**a**) FBG versus time; (**b**) serum insulin level and HOMA-IR index; (**c**) GTT and AUC index; (**d**) body weight versus time. Data are mean ± SD (*n* = 5). Compared with the control group, ## *p* < 0.01; Compared with the T2DM group, ** *p* < 0.01.

After being induced with STZ solution at week 4, the T2DM rat model was established successfully according to the FBG level and characteristics. The FBG, INS levels, and HOMA-IR in the T2DM group were significantly higher than those in the NC group, while compared with the T2DM group, rats received GFP-P showed lower levels of FBG, INS, and HOMA-IR (Figure 2a,b). Furthermore, in GTT testing, the blood glucose and calculated AUC increased in the T2DM group (Figure 2c), showing the successful establishment of T2DM model and the effect of GFP on glucose homeostasis together with the alteration of FBG, INS, and HOMA-IR.

Weight loss has proved to be an important characteristic of T2DM patients, which is also reflected in rat models. In our study, rats of the T2DM group weighed significantly less than those of the NC group, while rats of the GFP-P group weighed significantly more than those of the T2DM group (Figure 2d), indicating various preventive effects of GFP in T2DM rats. 

### 3.2. GFP Enhanced Intestinal Barrier Function in T2DM Rats

**Figure 3:**
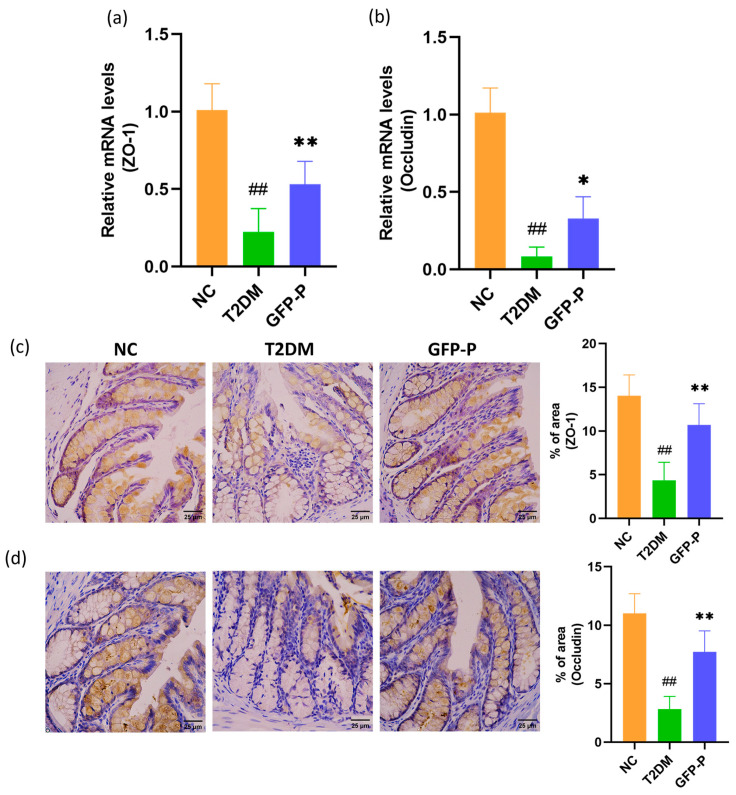
GFP enhanced intestinal barrier function in T2DM rats. (**a**,**b**) The relative expression levels of ZO-1 and Occludin mRNA were detected and analyzed by RT-qPCR; (**c**,**d**) the expression levels of ZO-1 and Occludin proteins were detected and analyzed by IHC (400×). Data are mean ± SD (*n* = 5). Compared with the control group, ## *p* < 0.01; compared with the T2DM group, * *p* < 0.05, ** *p* < 0.01.

It is well reported that intestinal mucosal barrier injury represents a significant mechanism underlying the onset and progression of T2DM, offering novel insights for its prevention and therapeutic interventions. Here, we examined the relative expression levels of biomarkers of intestinal mucosal barrier injury, ZO-1 and Occludin, in each group in mRNA and protein levels. And the results of both RT-qPCR and IHC showed that the expressions of ZO-1 and Occludin were significantly downregulated in the T2DM group but partially improved by GFP prevention (Figure 3a–d), indicating that GFP could enhance intestinal barrier function against T2DM in the rat model.

### 3.3. GFP Alleviated Intestinal Mucosal Barrier Injury and Inflammation in T2DM Rats

Impairment of the intestinal barrier in T2DM can precipitate a cascade of events, including the production of LPS and D-LA by the gut microbiota, as well as the release of DAO from the intestinal villi into the circulatory system via the compromised mucosal. Thus, the level of LPS, D-LA and DAO in the serum can serve as markers of intestinal mucosal barrier injury. 

**Figure 4:**
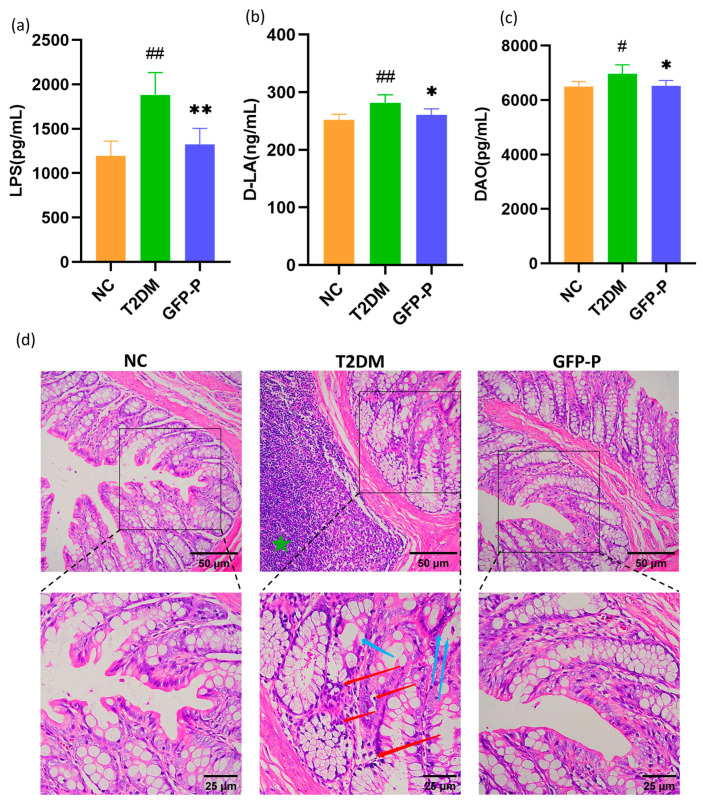
GFP alleviated intestinal mucosal barrier injury and inflammation in T2DM rats. (**a**–**c**) The expression levels of serum LPS, D-LA, and DAO were detected and analyzed by the ELISA kit; (**d**) the morphology of colon tissue was observed by HE (200×). Green star: lymphoid tissue hyperplasia in the T2DM group compared to the NC group; red arrow: inflammatory cell infiltration in the T2DM group compared to the NC group; blue arrow: gland atrophy and decreased goblet cells in the T2DM group compared to the NC group. Data are mean ± SD (*n* = 5). Compared with the control group, # *p* < 0.05, ## *p* < 0.01; Compared with the T2DM group, * *p* < 0.05, ** *p* < 0.01.

In this study, we demonstrated that T2DM rats acquired higher serum LPS, D-LA, and DAO levels but attenuated due to GFP pretreatment significantly through the ELISA detection (Figure 4a–c). Moreover, via HE staining of the colon from different groups, the morphology showed that the integrity of colonic mucosa and villi was impaired, and obvious glandular atrophy, deformation, and inflammatory infiltration were observed in the T2DM group. However, the morphology of colonic glands in the GFP-P group remained normal, and the inflammatory infiltration was also mild (Figure 4d), which demonstrates GFP prevention not only the ability to enhance intestinal barrier function but also alleviated inflammation in T2DM rats. 

### 3.4. GFP Ameliorated the Inflammation in Skeletal Muscle in T2DM Rats

**Figure 5:**
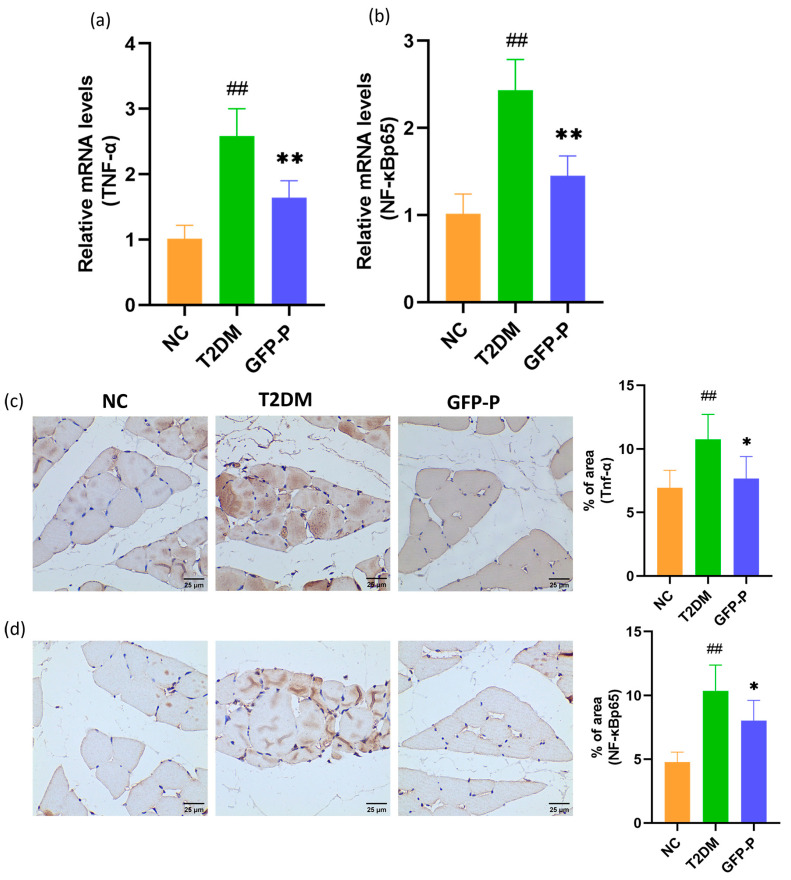
GFP ameliorated the inflammation in skeletal muscle in T2DM rats. (**a**,**b**) The expression of TNF-α, NF-κB p65 mRNA in gastrocnemius muscle was detected and analyzed by RT-qPCR; (**c**,**d**) the expressions of TNF-α, and NF-κB p65 proteins in gastrocnemius were detected and analyzed by IHC (400×). Data are mean ± SD (*n* = 5). Compared with the control group, ## *p* < 0.01; Compared with the T2DM group, * *p* < 0.05, ** *p* < 0.01.

The existence of the gut–muscle axis implies that inflammation resulting from intestinal barrier disruption may be conveyed to skeletal muscles through bacterial products released into the circulatory system and other means, thereby inducing an inflammatory response within the skeletal muscle and leading to atrophy. Hence, we further measured the relative levels of inflammatory indicators, TNF-α and NF-κB p65, in skeletal muscle. The RT-qPCR and IHC analyses revealed significantly elevated levels of TNF-α and NF-κB p65 (Figure 5a–d), indicating an exacerbated inflammatory state in the T2DM group, which could be also ameliorated by GFP administration.

### 3.5. GFP Prevented Skeletal Muscle Atrophy in T2DM Rats

**Figure 6:**
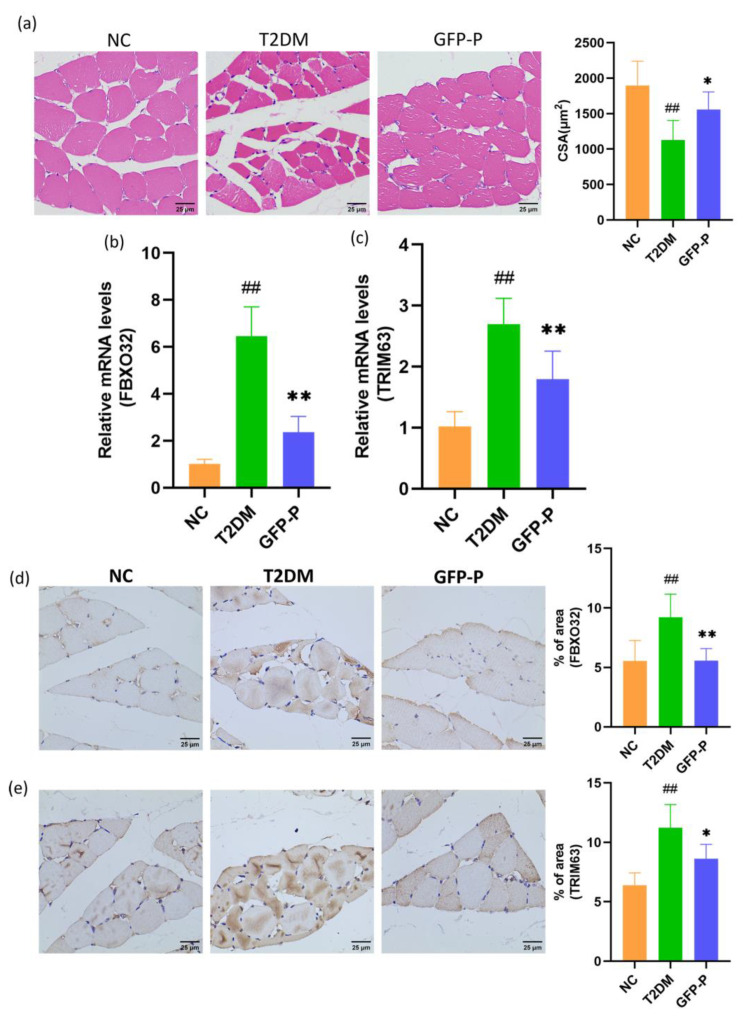
GFP prevented skeletal muscle atrophy in T2DM rats. (**a**) The cross-sectional area of gastrocnemius muscle fibrocytes was detected and analyzed by HE staining (400×); (**b**,**c**) the expression of FBXO32 and TRIM63 mRNA in gastrocnemius muscle was detected and analyzed by RT-qPCR; (**d**,**e**) the expression of FBXO32 and TRIM63 protein in gastrocnemius were detected and analyzed by IHC (400×). Data are mean ± SD (*n* = 5). Compared with the control group, ## *p* < 0.01; Compared with the T2DM group, * *p* < 0.05, ** *p* < 0.01.

Inflammation within skeletal muscle is a critical mechanism contributing to skeletal muscle atrophy in T2DM. HE staining and analysis of gastrocnemius muscle showed that the CSA of gastrocnemius muscle fibroblasts in the T2DM group was significantly reduced, and that of the GFP-P group was significantly improved (Figure 6a). Otherwise, the levels of atrophy markers, FBXO32 and TRIM63 (also known as Atrogin-1 and MURF1), in skeletal muscle were detected and analyzed by RT-qPCR and IHC, and it was found that FBXO32 and TRIM63 increased significantly in the T2DM group but partially attenuated in the GFP-P group (Figure 6b–e), indicating that GFP could prevent skeletal muscle atrophy in T2DM rats.

### 3.6. GFP Inhibited Inflammation and Prevented Skeletal Muscle Atrophy in T2DM Rats

**Figure 7:**
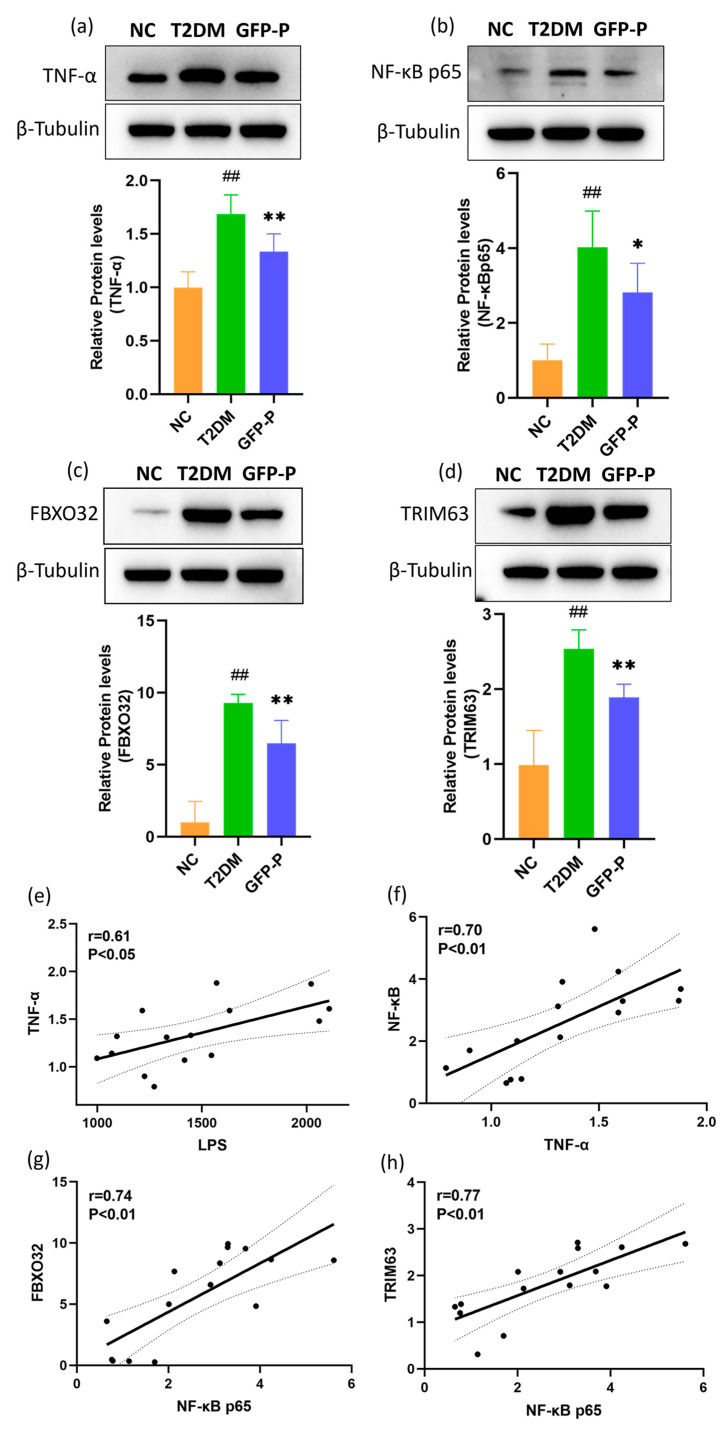
GFP inhibited inflammation and prevented skeletal muscle atrophy in T2DM rats (**a**–**d**) The levels of TNF-α, NF-κB p65, FBXO32, and TRIM63 protein were detected and analyzed by WB; (**e**) the correlation between serum LPS and gastrocnemius muscle TNF-α levels; (**f**) the correlation between gastrocnemius muscle TNF-α and NF-κB p65 levels; (**g**) the correlation between gastrocnemius NF-κB p65 and FBXO32 levels; (**h**) the correlation between of gastrocnemius muscle NF- κB p65 and TRIM63 levels. Dashed lines: 95% confidence interval; Data are mean ± SD (*n* = 5). Compared with the control group, ## *p* < 0.01; Compared with the T2DM group, * *p* < 0.05, ** *p* < 0.01.

To investigate the underlying mechanisms of the preventive effect of GFP on skeletal muscle atrophy, we measured the levels of TNF-α, NF-κB p65, FBXO32, and TRIM63 in skeletal muscle quantitatively by WB (Figure 7a–d), which firstly reconfirmed our conclusion obtained above that intensified inflammation and atrophy of skeletal muscle in T2DM could be prevented by GFP. 

Then, we further studied the correlation between serum LPS leaked from the damaged intestinal mucosal barrier and gastrocnemius TNF-α and between gastrocnemius TNF-α and NF-κB p65, key inflammatory regulators of muscle atrophy by inducing abnormal hydrolysis of muscle protein [28,29]. We found that there were positive correlations between LPS and TNF-α (r = 0.61, *p* < 0.05, Figure 7e) and between TNF-α and NF-κB p65 (r = 0.70, *p* < 0.01, Figure 7f). We next studied the correlation between NF-κB p65 and FBXO32 and between NF-κB p65 and TRIM63 in gastrocnemius muscle, and we found that there were positive correlations between NF-κB p65 and FBXO32 (r = 0.74, *p* < 0.01, Figure 7g) and between NF-κB p65 and TRIM63 (r = 0.77, *p* < 0.01, Figure 7h). These results indicate that GFP may prevent skeletal muscle atrophy by inhibiting inflammation in T2DM rats.

### 3.7. Molecular Docking of Monosaccharides Constituting GFP with Markers of Inflammation and Atrophy in Skeletal Muscle

**Figure 8:**
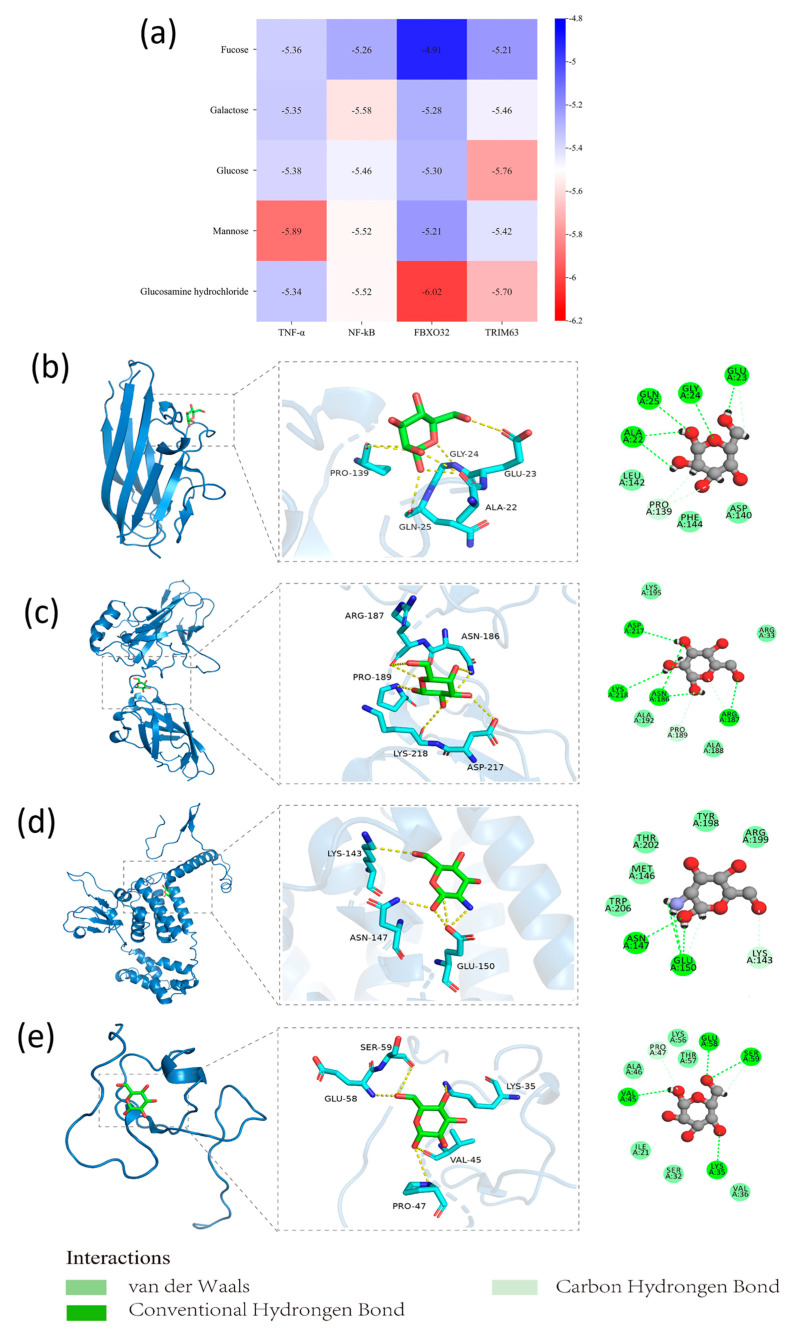
Binding free energy and molecular docking of monosaccharides of GFP with proteins participating skeletal muscle inflammation and atrophy. (**a**) Binding free energies; (**b**) mannose/TNF-α; (**c**) galactose/NF-kB; (**d**) glucosamine hydrochloride/FBXO32; (**e**) glucose/TRIM63.

The purified polysaccharide composition was composed of fucose, glucosamine hydrochloride, galactose, glucose, and mannose [26]. In order to further explore other potential mechanisms behind GFP in skeletal muscle atrophy of T2DM, we predicted the possibility of the interaction of five monosaccharides of GFP with TNF-α, NF-κB, FBXO32, and TRIM63 protein targets via molecular docking with MOE2019 software. According to the binding results, five monosaccharides could enter the binding domain of different target proteins, and the molecular docking energy range was −4.9107~−6.0204 kcal/mol. It is generally believed that the lower the binding free energy is, the more stable the binding of small molecules to the protein is, and a binding free energy <−5.0 kcal/mol indicates better binding activity. Among the 24 groups of docking, 23 groups had good binding activity, and 1 group had certain binding activity. Mannose/TNF-α (−5.8939 kcal/mol), galactose/NF-κB (−5.5793 kcal/mol), glucosamine hydrochloride/FBXO32 (−6.0204 kcal/mol), and glucose/TRIM63 (−5.7598 kcal/mol) had lower binding free energies, and visual analysis was carried out by pymol2.6.0 (Figure 8a).

The binding free energy of mannose/TNF-α was −5.8939 kcal/mol; multiple amino acid binding sites (Glu23, Gly24, Gln25, Ala22) of the TNF-α formed hydrogen bond interaction with mannose, and residues Pro139 and Glu23 formed carbon hydrogen interaction with the compound (Figure 8b). Meanwhile, galactose also showed good binding activity with NF-κB, and the active sites in NF-κB (Asp217, Lys218, Asn186, and Arg187) formed hydrogen bonds with galactose (Figure 8c). Compared with other monosaccharides, glucosaminamide hydrochloride had a lower binding free energy (−6.0204 kcal/mol) with FBXO32. The Glu150 and Asn147 sites in FBXO32 formed hydrogen bond interactions with glucosamine hydrochloride, and Lys143 and Glu150 formed carbon hydrogen interactions with glucosamine hydrochloride (Figure 8d). The combination of glucose and TRIM63 also had lower free energy (−5.7598 kcal/mol). The active sites of amino acids, such as Ser59, Glu58, Val45, and Lys35 in TRIM63, formed hydrogen bonds with glucose, and residues Pro47 and Ser59 formed C-H interactions with this compound (Figure 8e). These results also shed light on the potential mechanisms of GFP for the prevention of skeletal muscle atrophy in T2DM.

## 4. Discussion

In this study, we have successfully established a rat model of type 2 diabetes mellitus by employing a high-fat diet in combination with intraperitoneal injections of STZ. This model not only exhibited typical characteristics of T2DM, such as hyperglycemia, insulin resistance, and weight loss, but also mimicked various well-reported pathological features of T2DM patients, including impairment of intestinal barrier function [30,31], endotoxemia [32,33], increased systemic inflammation [34,35], and skeletal muscle atrophy [36,37]. The intestinal mucosal barrier injury in the T2DM model was thought to lead to the leakage of bacteria products from the intestinal cavity to the blood, especially endotoxin, which further enhances systemic inflammation including skeletal muscle. Furthermore, the intensified local inflammation resulted in the atrophy of skeletal muscle in the T2DM model. Yet, in addition to preventing hyperglycemic symptoms associated with T2DM, *Grifola frondosa* polysaccharide, as an important prebiotic, could alleviate systemic and local inflammation, reduce key inflammatory regulators TNF-α and NF-κB p65 in skeletal muscle resulting from T2DM-induced intestinal barrier dysfunction, and mitigate the consequent atrophy of skeletal muscle. Moreover, the monosaccharides constituting GFP have good binding activity with skeletal muscle inflammation and atrophy protein, which also indicates more potential mechanisms for the prevention of skeletal muscle atrophy. These findings suggest that GFP holds potential not only for managing hyperglycemia but also for addressing inflammatory and atrophic changes in skeletal muscle, offering a multifaceted approach to T2DM treatment.

The compromise of the intestinal barrier is not only a notable phenotype of diabetes but is also involved in its pathogenesis and progression. As a chronic metabolic disorder, diabetes always coincides with dysbiosis of the gut microbiota, which could lead to intestinal barrier destruction via various mechanisms, including the action of gut hormones and bacterial metabolites [38]. LPS, an important metabolic product of gut bacteria, can further translocate into the circulation upon impaired intestinal barrier [39,40]. Prolonged systemic exposure to even mildly elevated levels of LPS can trigger immune responses and activate inflammation pathways [41], which impedes insulin signaling, in turn, and contributes to the development of insulin resistance [42]. As reported, chronic inflammation is a hallmark of T2DM [8]. In our study, elevated serum LPS levels in the T2DM rat model were also significantly improved via GFP administration.

However, the existence of the gut–muscle axis, which means the interaction between the gut microbe and skeletal muscle, has made the mechanisms underlying the disease more complex [43]. As another significant pathological feature of diabetes, skeletal muscle atrophy has been well reported to be precipitated by metabolic abnormalities in diabetes, the underlying mechanisms of which are believed to be associated with a range of factors, especially inflammation [10]. Here, we have examined the TNF-α and NF-κB levels, the results of which indicate the inflammation pathways have been activated in the skeletal muscle of T2DM rats. The elevated TNF-α and NF-κB levels might be attributed to the increased LPS from the gut into serum according to the correlation analysis, which were significantly prevented by GFP treatment. The TNF-α and NF-κB further account for the intensive atrophy of skeletal muscle with increased FBXO32 and TRIM63 and reduced CSA, which is also improved by GFP prevention. In addition to TNF-α and NF-κB [44,45], an array of inflammatory mediators, including interleukin-6 (IL-6) [46], various chemokines, the signal transducer and activator of transcription 3 (STAT3) [46,47], and the suppressor of cytokine signaling 3 (SOCS3) [46], has been implicated in the pathogenesis of skeletal muscle atrophy in T2DM. The interconnections among these factors in the context of skeletal muscle atrophy and the impact of GFP on them remain to be explored in future studies.

Moreover, although we did not perform microbiome analysis, recent studies have demonstrated that GFP or some other polysaccharides affect gut barrier function through alterations in the gut microbiota and its metabolites [48]. Supplementation of a kind of GFP significantly increased the relative abundances of beneficial bacteria such as *L. acidophilus* and *Roseburia* [24]. The probiotic *L. acidophilus* not only reverses HFD-induced gut dysbiosis but also maintains intestinal barrier integrity, reduces metabolic endotoxemia, and inhibits the TLR4/NF-κB signaling pathway [49]. Another probiotic *Roseburia* can reduce gut-dysbiosis-induced mastitis by inhibiting bacterial translocation by producing butyrate in mice [50]. A variety of mushroom polysaccharides can enhance the intestinal barrier function by regulating the intestinal environment, increasing the proportion of a variety of beneficial bacteria, and promoting the production of metabolites such as short-chain fatty acids (SCFA) [48], which could inhibit NF-κB activation and TNF-α and other pro-inflammatory factors produced by neutrophils stimulated by LPS [51]. Future studies should aim to explore the detailed mechanisms of GFP’s action, its impact on the gut microbiota, and its potential clinical applications in larger and more diverse populations.

In conclusion, this study has demonstrated that GFP administration in the T2DM rat model could prevent skeletal muscle inflammation and consequent atrophy by enhancing intestinal barrier function and reducing systemic inflammation induced by endotoxin. Considering the actions of GFP in T2DM, it is believed that GFP holds potential as a therapeutic agent for the prevention of skeletal muscle atrophy associated with type 2 diabetes mellitus.

## Figures and Tables

**Table 1:** List of sequences of RT-PCR primers.

Gene	Primer	Sequence
ZO-1	Forward:	GGACGTTTATCGCCGCATTG
	Reverse:	TCCACGACCCGGAACACCT
Occludin	Forward:	AAAGCAGGGAAGGCGAAG
	Reverse:	TGTTGATCTGAAGTGATAGGTGG
TNF-α	Forward:	CTTCTCATTCCTGCTCGTGG
	Reverse:	TGATCTGAGTGTGAGGGTCTG
NF-κB p65	Forward:	CTACGAGACCTTCAAGAGCATC
	Reverse:	GATGTTGAAAAGGCATAGGGC
FBXO32	Forward:	CAACAGACTGGACTTCTCGAC
	Reverse:	GAAGTTCTTTTGGGCGATGC
TRIM63	Forward:	CCCCTTACAAAGCATCTTCCA
	Reverse:	TGTTTTCCTTGGTCACTCGG
β-actin	Forward:	CACTTTCTACAATGAGCTGCG
	Reverse:	CTGGATGGCTACGTACATGG

## Data Availability

Data are contained within the article.
